# Identification of transformer fault based on dissolved gas analysis using hybrid support vector machine-modified evolutionary particle swarm optimisation

**DOI:** 10.1371/journal.pone.0191366

**Published:** 2018-01-25

**Authors:** Hazlee Azil Illias, Wee Zhao Liang

**Affiliations:** Department of Electrical Engineering, Faculty of Engineering, University of Malaya, Kuala Lumpur, Malaysia; Northeast Normal University, CHINA

## Abstract

Early detection of power transformer fault is important because it can reduce the maintenance cost of the transformer and it can ensure continuous electricity supply in power systems. Dissolved Gas Analysis (DGA) technique is commonly used to identify oil-filled power transformer fault type but utilisation of artificial intelligence method with optimisation methods has shown convincing results. In this work, a hybrid support vector machine (SVM) with modified evolutionary particle swarm optimisation (EPSO) algorithm was proposed to determine the transformer fault type. The superiority of the modified PSO technique with SVM was evaluated by comparing the results with the actual fault diagnosis, unoptimised SVM and previous reported works. Data reduction was also applied using stepwise regression prior to the training process of SVM to reduce the training time. It was found that the proposed hybrid SVM-Modified EPSO (MEPSO)-Time Varying Acceleration Coefficient (TVAC) technique results in the highest correct identification percentage of faults in a power transformer compared to other PSO algorithms. Thus, the proposed technique can be one of the potential solutions to identify the transformer fault type based on DGA data on site.

## Introduction

Power transformer is a vital component in power system networks. Failure of power transformers can interrupt power system network operation. Thus, any fault in a transformer should be detected early. Electrical fault in a transformer occurs at high voltage and will eventually cause physical damage to the conductor and insulator of the transformer, leading to the reduction of power quality, blackouts and fire, causing a substantial propriety loss. Damage in a transformer is difficult to be repaired and the transformer replacement is very costly and requires a lot of resources. Therefore, early detection of transformer fault is imperative in the operation and maintenance process of power system networks. This is to ensure correct transformer oil maintenance, cost reduction and good quality of electricity supply to power systems.

Exposure to electrical and thermal stress causes molecules of hydrocarbon of the mineral oil decomposition and hydrogen and carbon are formed. Under high voltage, these gases can react with each other to form various gases such as ethylene (C_2_H_4_), methane (CH_4_), acetylene (C_2_H_2_), hydrogen (H_2_), and ethane (C_2_H_6_). Previous studies have revealed that as temperature increases, gases are generated in the order of H_2_, CH_4_, C_2_H_6_, C_2_H_4_ and C_2_H_2_ [1]. Overheating and corona typically lead to the insulation material decomposition [2]. Decomposition of insulation materials such as paper and cellulose in a transformer can be analysed according to the amount of carbon dioxide (CO_2_) and carbon monoxide (CO) present in the transformer oil. This is due to depolymerisation causes breaking of glucose ring chains in the paper. CO_2_, CO and H_2_O are emitted due to the presence of oxygen atoms in the cellulose materials.

The existing methods of detecting the incipient fault in transformer oil are IEC ratio method, Rogers ratio method, key gas method and Doernenburg ratio method. However, gas ratio usage is based on experience through correlating amount of gas concentration and the type of fault. Each of this method has disadvantages, rigorous borderline and hidden relationship [3]. For example, key gas method requires a gas of significant amount to exist in the oil sample and is unable to give a conclusion in some cases. Thus, improvement on the accuracy of the existing method to identify the fault type in transformer oil is important. Utilisation of artificial intelligence and optimisation methods has shown convincing results in power transformer modelling [4, 5]. Artificial intelligence has also been widely applied in transformer fault diagnosis and in power systems [6–9].

In An-xin Zhao’s work [10], four combustible gases and six combination of gas ratios, which correlate to six fault types were considered. The accuracy rates were calculated for 117 cases by different methods. From this work, the best methods of thermal and electrical fault diagnosis are found to be Duval Triangle and KimSW methods [11]. Each method achieves accuracy higher than 55%. However, the accuracy according to the total number of cases shows various results. Since there are many cases undistinguishable, the accuracy reduces less than 55% for Roger and Doernenburg ratio methods.

Based on the work in [12], rules of IEC Code and Roger’s ratio were modified to enhance the accuracy of the current version based on 320 samples with its actual fault type. The samples were categorised into six fault types, which are high thermal fault (Tl), low thermal fault, medium thermal fault (T2), low energy discharge (Dl), high energy discharge (D2) and partial discharge (PD). The overall accuracy of the modified Roger’s four ratios increases from 45.62% to 75.62%. For IEC Code method modification, significant variation was observed after modification for all transformer fault types, where the overall accuracy increases from 62.81% to 79.38%. Hence, the new version of Roger’s four ratios and IEC Standard Code methods have the ability to identify the transformer fault with higher accuracy than the unmodified version.

In [13], genetic algorithm (GA) and support vector machine (SVMG) were employed on power transformer fault identification, where GA was applied to optimise SVM parameters. SVM classifiers were employed to identify the no-fault state, high-energy discharge, low-energy discharge, high-temperature overheating, low- and mid-temperature overheating. From the results obtained, it was found that SVMG achieves better diagnosis results compared to the IEC method, back propagation neural network and SVM alone. Other works have also shown that optimisation method can improve the performance of the system significantly [14, 15].

PSO and SVM were employed to predict dissolved gas contents in power transformers [16]. PSO was used to optimise the SVM parameters to avoid under or over-fitting of the SVM model. It was found that the proposed method achieves better forecasting accuracy than grey model and artificial neural network (ANN) under small number of samples. In [17], SVM was employed in transformer fault diagnosis on DGA data. One-against-all, one-against-one and binary decision tree were applied. From the results, the highest accuracy was achieved by SVM one-against-one method, which is 92%.

Identification of transformer fault was also proposed using different PSO and ANN techniques [18]. The PSO optimisation methods employed were traditional PSO, evolutionary PSO and iteration PSO. From the fault identification algorithm, ANN-EPSO results in the highest correct identification percentage of transformer fault than the previously reported works and existing DGA methods. In [19], ANN was employed to classify transformer condition based 3 combustible gas ratios. Three ratios of combustible gases were applied as the input. From this method, the validation and test set errors have the same characteristics and no overfitting.

Although SVM has been applied widely in classification of gas ratio, limited amount of work has been reported on transformer fault identification using SVM combined with various optimisation techniques. It is believed that there is room for improvement in combining SVM with various optimisation techniques in identification of power transformer faults. Thus, this work proposes a hybrid SVM with different particle swarm optimisation (PSO) techniques to identify the transformer faults. Feature selection using stepwise regression was also applied prior to the SVM training for data reduction. This was done to enable only meaningful sets of data are used in the training of SVM. The effectiveness of each method was evaluated by comparing the results with the actual fault and existing methods, unoptimised SVM and previous works using ANN. A better result of the proposed method than the previously reported work will indicate that this method can be an alternative solution for transformer fault diagnosis on site.

This work is organised as follows. Section 1 is the introduction of this work, which includes problem statement and review of published related works. Section 2 describes the support vector machine and feature selection method used in this work. Section 3 explains different types of particle swarm optimisation (PSO) techniques. In Section 4, all findings and analysis are presented. Finally, Section 5 is the conclusions, which presents a summary of the key findings.

## Intelligent classifier and feature selection

### Support vector machine (SVM)

SVM was introduced by Vapnik and Chervonenkis, which was derived from statistical learning theory [20–22]. SVM is useful in dealing with non-linear separable cases [23]. SVM is a powerful technique used for data classification and data prediction [24]. The training of SVM was performed where the classified vector dimension has no distinct influence on the SVM performance. Thus, SVM has the capability of handling a very large feature space, giving SVM higher efficiency compared to other classification techniques especially when dealing with large amount of classification data. Since the past, SVM has been employed widely to solve many practical problems in various fields [25–28]. The flexibility of SVM makes it beneficial in transformer fault identification due to the number of features that forms the fault diagnosis basis is not limited.

In this work, radial basis function (RBF) kernel was employed. Two parameters in SVM were optimised by different PSO algorithms in this work, *σ* and *c*. *σ* is a RBF kernel parameter while *c* represents the misclassification parameter and determines the trade-off between the size and margin of slack variables [29]. A large *c* means a higher penalty for non-separable points. A small *c* is an indication that an under-fit process is occurring. Both *c* and *σ* are important as the combination of these two parameters will influence the percentage of correct fault identification by SVM.

### Stepwise regression

Feature selection enables the selection of significant data for training and testing of transformer fault type using SVM. This technique prevents the use of a large amount of redundant information and insignificant features in the dataset, which often leads to greater complexity, longer run time and lower accuracy [30]. In this work, stepwise regression technique was employed to simplify the model to be interpreted by the SVM and to reduce the chance of overfitting, giving a shorter run time while maintaining high percentage of correct fault identification.

Stepwise regression is a regression-based algorithm with automatic filtering features [31]. It is utilised to select the important features of the training and testing dataset [32]. Hence, the most significant statistical features are selected by the algorithm. Stepwise regression analyses the significance of the model in term of statistical using partial F-statistic. Enabling users to determine the type of gases has the greatest influence on the fault type identification.

In this work, the forward selection characteristic of stepwise regression was utilised. Each term was removed from or added to the input feature vector with reference to the *p-value* of exiting or entering input data. Null-hypothesis test probability is represented by *p-value* with *α* tolerance for a term addition and *β* tolerance for a term removal. In this work, each input data represents a type of gas obtained from DGA. The input data were selected based on the *p-value* resulted from stepwise regression.

### Input and output data for SVM classifier

Different PSO techniques were employed to optimise the SVM to identify transformer fault type using DGA data obtained from an electrical utility in Malaysia. The parameters of each method which give the highest percentage of correct fault identification and yield the shortest test time were selected for the optimization objective. Feature selection of training and testing data were carried out using stepwise regression to reduce the run time while maintaining a high percentage of correct fault identification.

Numbers of run were tested to examine the performance of each method. The results were compared between different PSO methods with the existing DGA method, which is IEC 60599 method. 400 data from the actual site diagnosis were used in this work. The raw data were classified into 64 cases of low and high intensity discharge each, 72 cases of thermal fault and 200 cases without fault. Table 1 shows the input (dissolved gas) and output data (fault type) used for SVM training and testing. Seventy percent of the overall data was used to train the SVM while for testing purpose, the remaining data were used.

**Table 1:** Data for input and output data for SVM.

Input data (dissolved gas)	Output data (fault type)
Concentration of H_2_ (Hydrogen)	
Concentration of C_2_H_2_ (Acetylene)	Electrical Fault–Low energy
Concentration of CH_4_ (Methane)	Electrical Fault–High energy
Concentration of CO (Carbon Monoxide)	Thermal Fault
Concentration of C_2_H_6_ (Ethane)	No fault
Concentration of C_2_H_4_ (Ethylene)	

## Particle swarm optimisation techniques

### Conventional PSO

**Fig 1:**
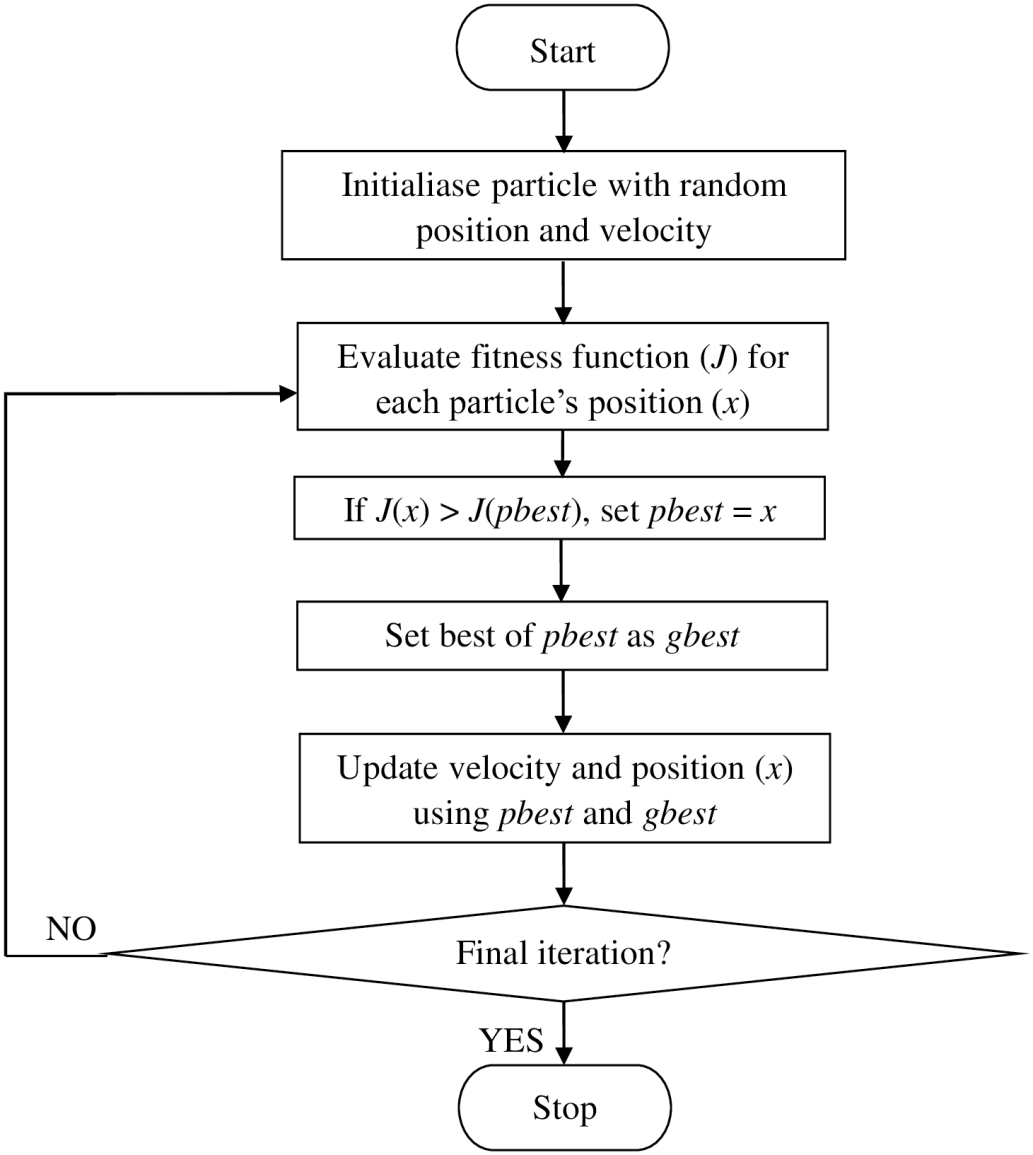
Flowchart of SVM-PSO algorithm.

PSO is an evolutionary computational algorithm to solve non-linear optimisation problems [30, 33–36]. The advantage of PSO is the PSO parameters can be empirically adjusted to optimise its performance for the best results. Fig 1 shows a PSO algorithm flowchart to optimise the performance of SVM. First, swarms were initialised randomly with velocity and initial position of every particle. The swarms are the SVM parameters, *c* and *σ*. Then, the fitness for every particle, which is the accuracy of SVM, was calculated with the initial position and velocity. After that, the fitness of every each particle was compared with its personal best *pb*_*id*_^*k*^. When the new fitness is better than *pb*_*id*_^*k*^, it is set as *pb*_*id*_^*k*^ and *X*_*id*_^*k*^ or the particle’s current position. The overall best fitness is taken as the global best *gb*_*d*_^*k*^. Next, the updated position and velocity of every particle were calculated using

$$X_{id}{}^{k+1}=X_{id}{}^{k}+V_{id}{}^{k+1}$$ (1)

$$V_{id}{}^{k+1}=wV_{id}{}^{k}+c_{2}r_{2}(gb_{d}{}^{k}-X_{id}{}^{k})+c_{1}r_{1}(pb_{id}{}^{k}-X_{id}{}^{k})$$ (2)

where *X*_*id*_^*k*+1^ is the new position of particle *i*, *X*_*id*_^*k*^ is the previous position of particle *i* at *k*-th iteration, *V*_*id*_^*k*+1^ is the new particle *i* velocity in *d* dimension, *V*_*id*_^*k*^ is particle *i* velocity at iteration *k*, *r*_2_ and *r*_1_ are randomly generated number between 0 and 1 and *c*_2_ and *c*_1_ are the acceleration factors. In this work, the domains of *X*_*i*_ are two SVM parameters, *c* and *σ*. The inertia weight, *w* is determined using

$$w=w_{\text{max}}-k\left(\frac{w_{\text{max}}-w_{\text{min}}}{k_{\text{max}}}\right)$$ (3)

where *k*_*max*_ is the final iteration, *w*_*min*_ is the minimum weight and *w*_*max*_ is the maximum weight. If any particle moves out of the bounds, its position is set to the upper or lower boundary. The algorithm was halted when the final number of iteration was reached.

### Iteration particle swarm optimisation (IPSO)

IPSO incorporates the iteration best, *Ib* in the existing PSO algorithm. *Ib* is the optimum value of fitness function by a particle in an iteration. IPSO improves the quality of the solutions and efficiency of the existing PSO [37]. The new velocity equation for each particle in IPSO is given by

$$V_{id}{}^{k+1}=wV_{id}{}^{k}+c_{3}r_{3}(Ib_{d}{}^{k}-X_{id}{}^{k})+c_{2}r_{2}(gb_{id}{}^{k}-X_{id}{}^{k})+c_{1}r_{1}(pb_{id}{}^{k}-X_{id}{}^{k})$$ (4)

where *Ib*_*d*_^*k*^ is the best fitness obtained by any particle at *k*-th iteration and *c*_3_ is the stochastic acceleration weight to attract every particle towards *Ib*_*d*_^*k*^. *c*_3_ is calculated using

$$c_{3}=c_{1}[1-\text{exp}(-kc_{1})]$$ (5)

### Evolutionary particle swarm optimisation (EPSO)

EPSO is a meta-heuristic algorithm that combines the concept of PSO and Evolution Strategies (ES). This algorithm was introduced by Miranda [38]. EPSO introduces a mutation operation into PSO, causing each particle to have its weight mutated. EPSO provides a procedure of explicit selection with self-adapting properties in its parameters. This allows the solution with superior characteristic to be passed down from a generation to the next generation. Hence, the equation of the updated velocity is modified to

$$V_{id}{}^{k+1}=w_{i0}{}^{*}V_{id}{}^{k}+w_{i1}{}^{*}(pb_{id}{}^{k}-X_{id}{}^{k})+w_{i2}{}^{*}(gb_{id}{}^{*}-X_{id}{}^{k})$$ (6)

where *w*_*i*0_*, *w*_*i*1_* and *w*_*i*2_* are the mutated weight and *gb*_id_* is the mutated global best position. *gb*_id_*, *w*_*i*0_*, *w*_*i*1_* and *w*_*i*2_* are calculated using

$$gb_{id}{}^{*}=gb_{id}{}^{k}+\tau \prime N$$ (7)

$$w_{ij}{}^{*}=w_{ij}+\tau N$$ (8)

where *j* = 0, 1 and 2. *N* is a random number between 0 and 1 with Gaussian distribution, variance equals to 1 and zero mean. *τ* is the learning dispersion parameter and *τ*’ is the noise dispersion parameter. *τ* and *τ*’ are the learning parameters introduced in EPSO algorithm to facilitate the search for the optimum values for the desired parameters.

### Modified PSO (MPSO)-Time varying acceleration coefficient (TVAC)

In MPSO-TVAC, the main function of this technique is to avoid search form converging prematurely and to enhance the convergence rate to the optimum global solution at the later search stage [39–41]. A new term *rb* is added into the velocity equation of PSO to improve the PSO robustness by providing additional information for every particle, where *rb* is selected from other particles’ *pb* randomly. Hence, the premature convergence can be avoided and particles’ movement is diversified. The new updated velocity for MPSO-TVAC is given by

$$V_{id}{}^{k+1}=wV_{id}{}^{k}+c_{3}r_{3}(rb_{id}{}^{k}-X_{id}{}^{k})+c_{2}r_{2}(gb_{id}{}^{k}-X_{id}{}^{k})+c_{1}r_{1}(pb_{id}{}^{k}-X_{id}{}^{k})$$ (9)

where

$$c_{1}=c_{1i}+\left(k/k_{\text{max}}\right)\left(c_{1f}-c_{1i}\right)$$ (10)

$$c_{2}=c_{2i}+\left(k/k_{\text{max}}\right)\left(c_{2f}-c_{2i}\right)$$ (11)

$$c_{3}=c_{1}\left[1-\text{exp}\left(-c_{2}k\right)\right]$$ (12)

*c*_3_ is the TVAC for *rbest*, *c*_*2f*_ and *c*_*2i*_ are the final and initial values of the social coefficient respectively, *c*_2_ is the social coefficient and *c*_1_ is the cognitive coefficient. *c*_*1f*_ and *c*_*1i*_ are the final and initial cognitive coefficient values respectively.

### Modified EPSO (MEPSO)-TVAC

By introducing time varying acceleration coefficient (TVAC), the updated velocity for EPSO technique is improved. The updated velocity equation is obtained by

$$V_{id}{}^{k+1}=w_{i0}{}^{*}V_{id}{}^{k}+w_{i1}{}^{*}(pb_{id}{}^{k}-X_{id}{}^{k})+w_{i2}{}^{*}(gb_{id}{}^{*}-X_{id}{}^{k})+w_{i3}{}^{*}(rb_{id}{}^{*}-X_{id}{}^{k})$$ (13)

where

$$w_{i0}{}^{*}=w+N\tau$$ (14)

$$w_{i1}{}^{*}=c_{1}+N\tau$$ (15)

$$w_{i2}{}^{*}=c_{2}+N\tau$$ (16)

$$w_{i3}{}^{*}=c_{3}+N\tau$$ (17)

*w*_*i*1_*, *w*_*i*2_* and *w*_*i*3_* are the mutated weight.

## Results and discussion

### Optimisation of SVM without stepwise regression

Table 2 shows the results obtained using different PSO algorithms from SVM that have been performed in this work. For each algorithm, different combinations of PSO parameters were tested, which include varying *c*_1_, *c*_2_, *w*_*max*_ and *w*_*min*_ to obtain the best accuracy results of SVM. Each combination of *c*_1_ and *c*_2_ were varied from 0.1 to 2.0 with a step size of 0.1. Each test was repeated for 100 times to obtain the average result. The population size was set to 50. From Table 2, it can be seen that each algorithm yields different parameters, average SVM accuracy and run time, number of convergence at first iteration and best *c* and *σ*. The accuracy of the SVM was calculated based on the percentage of correct fault type identification.

**Table 2:** Results using different PSO algorithms for SVM without stepwise regression.

Algorithm	SVM-PSO	SVM-IPSO	SVM-EPSO	SVM-MPSO-TVAC	SVM-MEPSO-TVAC
Best parameters	*c*_1_ = 0.6	*c*_1_ = 1.5	*c*_1_ = 1.3		
	*c*_2_ = 0.6	*c*_2_ = 1.7	*c*_2_ = 1.9	*w*_*max*_ = 0.9	*w*_*max*_ = 0.9
	*w*_*max*_ = 0.9	*w*_*max*_ = 0.9	*w*_*max*_ = 0.9	*w*_*min*_ = 0.4	*w*_*min*_ = 0.4
	*w*_*min*_ = 0.4	*w*_*min*_ = 0.4	*w*_*min*_ = 0.4		
Average accuracy (%)	98.88	99.00	99.01	99.10	99.50
Average run time (s)	74.3678	75.2416	80.9249	88.5753	90.8578
Number of convergence at first iteration	27 / 100	75 / 100	83 / 100	91 / 100	93 / 100
	(27%)	(75%)	(83%)	(91%)	(93%)
Best *c*	1.5741	1.3692	1.6083	1.5810	1.6261
Best *σ*	0.4637	0.5345	0.3399	0.3896	0.4738

From the results obtained, the proposed SVM-MEPSO-TVAC technique yields the highest accuracy with an average accuracy of 99.50% and with an average run time of 90.8578s. It is followed by SVM-MPSO-TVAC with 99.10% accuracy, SVM-EPSO with 99.01% accuracy, SVM-IPSO with 99.00% accuracy and finally SVM-PSO with 98.88% accuracy. From 100 runs, the lowest number of run which converges at first iteration was achieved by SVM-PSO, which is 27 or 27% while the highest number of run which converges at first iteration is from SVM-MEPSO-TVAC, with 93 runs or 93%. When the percentage of convergence at first iteration is higher, this indicates that the algorithm can be run with less maximum iteration, which will reduce its run time. The shortest average run time is SVM-PSO while the longest average run time is SVM-MEPSO-TVAC.

The main advantages of PSO are simple, computation efficient and easy to be implemented. This is why the average run time is the fastest, as shown in Table 2. In PSO, the search towards the optimal solution of the parameter is guided by 2 stochastic acceleration components, the social and cognitive components. Proper setting of these components is vital to yield accurate and efficient search towards the optimum solution. If the cognitive component is higher than the social component, of individuals will be wandering excessively through the searching space. However, if the social component is higher than the cognitive component, particles will rush toward a local optimum prematurely. Hence, improvement on PSO algorithm, such as allowing the automated search of the stochastic acceleration term weighting and introduction of mutation feature has shown enhancement on the average accuracy of the SVM.

### Optimisation of SVM with stepwise regression

Stepwise regression was applied on the input and output data to select gases obtained from DGA with the most dominant characteristic in transformer fault identification. The results obtained after stepwise regression are shown in Table 3. It can be seen that the data used for SVM training and testing displays similar characteristics. The *p-value* tests the null hypothesis, which the coefficient equals to zero. A predictor with low *p-value* as displayed by CO with a value of 1.0214×10^−36^ is a meaningful addition to the model of the selected features. A smaller *p-value* possessed by the gas reflects that the DGA data for that particular gas has a stronger association with the transformer fault type. In this work, stepwise regression shows that the type of gas in the order of decreasing significance towards the identification of transformer fault type for training data and testing data is CO, H_2_, C_2_H_6_, CH_4_, C_2_H_2_ and finally C_2_H_4_.

**Table 3:** Result for feature selection using stepwise regression.

Type of gas	Training data			Testing data		
	*p-value*	Regression coefficient (×10^−3^)	Standard error (×10^−3^)	*p-value*	Regression coefficient (×10^−3^)	Standard error (×10^−3^)
Hydrogen,H_2_	5.6278×10^−21^	0.1692	0.014007	5.3723×10^−21^	0.1684	0.013927
Acetylene,C_2_H_2_	0.2335	-0.0266	0.022164	0.2251	-0.0269	0.022028
Methane, CH_4_	0.1682	0.0016	0.001118	0.1769	0.0016	0.001504
Carbon monoxide,CO	1.0214×10^−36^	0.0071	0.000349	1.0979×10^−36^	0.0071	0.000349
Ethane, C_2_H_6_	0.0397	-0.0155	0.007428	0.0377	-0.0156	0.007420
Ethylene, C_2_H_4_	0.9550	-0.0005	0.009492	0.9325	-0.0008	0.009491

Standard error is important to access the strength of the relationship between the model made up of selected features and the response variable. Also, standard error accesses the validity of the *p-values* as it represents the distance that the observed values fall from the regression line in mathematical form. A smaller standard error equates a better response as the model obtained gives observations of the response variable, which is the fault type closer to the fitness line. From Table 3, it is observed that there is a small value of standard error with values less than 10^−4^ for all types of gases in training and testing data. With *p-value* and standard error showing small values, it can be deduced that the selected features have a strong relationship with the response variables, where smaller values display a stronger relationship.

The regression coefficient in Table 3 shows the change of mean in the response parameter for a unit of change in the specific predictor parameter while the other predictors are kept constant. For example, in training data, the coefficient for H_2_ is 0.1692×10^−3^. This indicates that for every unit addition in the concentration of H_2_, it can be expected that the overall result of the response variable increases by 0.1692×10^−3^.

Based on the stepwise regression results as shown in Table 3, for 3 gas input data, H_2_, CO and C_2_H_6_ were used as the input data for testing and training of SVM. These 3 gases are ranked at the three lowest *p-values*. For 4 gas input, H_2_, CO, CH_4_ and C_2_H_6_ were taken as the input gas while for 5 gas input, H_2_, CO, CH_4_, C_2_H_2_ and C_2_H_6_ were taken as the input gas. Table 4 shows the type of gases for training and testing data according to stepwise regression for different number of gas input. By decreasing the number of input data applied to SVM, the size of the kernel matrix and complexity of the computing can be reduced, hence reducing the test time.

**Table 4:** Type of gases for training and testing data according to stepwise regression (√ means included, X means excluded).

Type of gas	Training data				Testing data			
	Number of gas input				Number of gas input			
	*n* = 3	*n* = 4	*n* = 5	*n* = 6	*n* = 3	*n* = 4	*n* = 5	*n* = 6
Hydrogen,H_2_	√	√	√	√	√	√	√	√
Acetylene,C_2_H_2_	X	X	√	√	X	X	√	√
Methane, CH_4_	X	√	√	√	X	√	√	√
Carbon monoxide,CO	√	√	√	√	√	√	√	√
Ethane, C_2_H_6_	√	√	√	√	√	√	√	√
Ethylene, C_2_H_4_	X	X	X	√	X	X	X	√

Table 5 shows results using MEPSO-TVAC and MPSO-TVAC algorithms on SVM by using different number of gas input for 100 runs. These two algorithms were selected to be tested due to their average SVM accuracy is two highest values according to Table 2. From Table 5, when 3 gas input was used, the accuracy for SVM-MPSO-TVAC and SVM-MEPSO-TVAC is 99.02% and 99.45% each with average run time of 53.1552s and 55.0159s respectively. The number of run which converges at first iteration is 97 and 100 out of 99 for SVM-MPSO-TVAC and SVM-MEPSO-TVAC respectively. Comparing with 6 gas input data, the average run time for SVM-MPSO-TVAC and SVM-MEPSO-TVAC is 88.5753s and 90.8578s respectively but the average SVM accuracy is 99.10% and 99.50% for SVM-MPSO-TVAC and SVM-MEPSO-TVAC respectively.

**Table 5:** Results using different PSO algorithms for SVM with stepwise regression.

Number of gases used	3		4		5		6	
Technique	SVM-MPSO-TVAC	SVM-MEPSO-TVAC	SVM-MPSO-TVAC	SVM-MEPSO-TVAC	SVM-MPSO-TVAC	SVM-MEPSO-TVAC	SVM-MPSO-TVAC	SVM-MEPSO-TVAC
Average accuracy (%)	99.02	99.45	99.05	99.47	99.05	99.48	99.10	99.50
Average run time (s)	53.1552	55.0159	65.9056	72.9114	74.6939	76.9592	88.5753	90.8578
Number of convergence at first iteration	97 out of 100	99 out of 100	94 out of 100	99 out of 100	91 out of 100	93 out of 100	91 out of 100	93 out of 100
	(97%)	(99%)	(94%)	(99%)	(91%)	(93%)	(91%)	(93%)
Best c	1.2324	1.2453	1.2434	1.5000	1.3676	1.5481	1.5810	1.6261
Best *σ*	0.2246	0.2280	0.2404	0.3059	0.3923	0.5285	0.3896	0.4738

Referring to Table 5, when the number of gas input used is higher, the percentage of correct fault detection (average SVM accuracy) improves with a small percentage only. However, it is obvious that the average run time increases significantly when the number of gas input used is higher due to more data are used. The number of convergence at first iteration decreases slightly when more gas input is used because the number of particles in PSO algorithm is higher. Therefore, this shows that using data reduction by stepwise regression for SVM training and testing data, the run time can be reduced significantly without affecting the accuracy results of the SVM.

From Table 5, comparison between SVM-MPSO-TVAC and SVM-MEPSO-TVAC shows that SVM-MEPSO-TVAC has higher number of convergence at first iteration and average SVM accuracy than SVM-MPSO-TVAC for different number of gas input data. However, the average run time slightly longer for SVM-MEPSO-TVAC compared to SVM-MPSO-TVAC when different number of gas input was used. Mutation in the particle weight is a critical improvement in SVM-MEPSO-TVAC over SVM-MPSO-TVAC.

### Comparison with the previous works

The proposed hybrid SVM with various PSO techniques employed in this work were compared with the existing works in literature on the transformer fault identification according to dissolved gas analysis data. Table 6 shows the comparison between the proposed methods and previously developed works. It is observed that the proposed SVM-MEPSO-TVAC method results in the highest accuracy with 99.50% compared to other reported existing works.

**Table 6:** Comparison between different methods.

Method	Correct fault identification (%)
IEC method	75.00
Unoptimised SVM	97.00
Proposed SVM-PSO	98.88
Proposed SVM-IPSO	99.00
Proposed SVM-EPSO	99.01
Proposed SVM-MPSO-TVAC	99.10
Proposed SVM-MEPSO-TVAC	99.50
ANN-PSO [18]	96.00
ANN-IPSO [18]	97.00
ANN-EPSO [18]	98.00
Self-organizing polynomial network (SOPN) [42]	97.68
Genetic wavelets network (GWN) [43]	96.19
Support Vector Machine (SVM) [44]	92.00
Genetic programming-*k*-neural network (GP-KNN) [45]	92.11
Rough Set Theory [46]	81.25
Immune Neural Network [47]	86.30
Evolutionary programming-artificial neural network (EPANN) [48]	95.00
Artificial neural network-expert system (ANNEPS) [49]	90.95

Referring to Table 6, the IEC method yields only 75.00% accuracy. IEC method is based on the existing dissolved gas analysis (DGA) technique, which utilises three different ratios of the gas. These include CH_4_/H_2_, C_2_H_2_/C_2_H_4_ and C_2_H_4_/C_2_H_6_. This method does not depend on specific gas concentrations to exist in the transformer but it is used only when the normal limits of the individual gases have been exceeded. Hence, it is not suitable to be used in all condition of gas concentration. This is why the accuracy obtained using the IEC method in this work is low.

The accuracy of the unoptimised SVM, proposed SVM-PSO, SVM-IPSO, SVM-EPSO and SVM-MPSO-TVAC is 97.00%, 98.88%, 99.00%, 99.01% and 99.10% respectively. These are the results obtained from this work. Comparing these results with the IEC method, it shows a significant improvement in the percentage of correct prediction of the fault type, which is over 20%. Referring to results reported in [18], which applied artificial neural network (ANN) as the classifier, the accuracy results as in Table 6 show that the proposed methods using SVM is generally higher than using ANN. The accuracy result using ANN-PSO, ANN-IPSO and ANN-EPSO is 96.00%, 97.00% and 98.00% respectively.

SVM is a supervised learning machine, which uses mainly the kernel method. SVM locates the decision function from a set of labelled training data. The decision function is made by hyperplane, which segregates the training data into two groups. The solution of the classification learning problem is obtained in terms of a subset of training data, also known as support vectors. An optimal hyperplane will be selected from all the separating hyperplanes, whereby the margin of separation between itself and any training point is of maximum value. This is why SVM yields better results than ANN and the IEC method. Therefore, referring to the results shown in Table 6, it has been demonstrated that the hybrid SVM with MEPSO-TVAC algorithm proposed in this work can be an alternative solution for diagnosis of transformer fault type based on DGA in industrial practice.

## Conclusions

In this work, hybrid support vector machine (SVM) with modified particle swarm optimisation (PSO) algorithms has been successfully proposed in optimising the SVM performance. In the proposed method, SVM was successfully employed to determine the transformer fault type and different algorithms of PSO were employed to optimise the SVM parameters. From results comparison, the proposed hybrid SVM with modified EPSO-Time Varying Acceleration Coefficient (TVAC) yields the best performance in terms of correct identification percentage of transformer fault compared to the current DGA method, unoptimised SVM and the reported existing works. After feature selection using stepwise regression was applied, it was found that there is a significant reduction in the training and testing time but the accuracy of SVM-MEPSO-TVAC is still high when less number of gas input was used. The proposed SVM-MEPSO-TVAC also achieves highest number of convergence at first iteration than other SVM-PSO algorithms. Hence, the proposed SVM-MEPSO-TVAC method combined with feature selection using stepwise regression can be proposed as an alternative solution for power transformer diagnosis.

## Supporting information

S1 Table Dissolved gas analysis (DGA) data. (DOCX)
