# Supplementary material for: Identification of transformer fault based on dissolved gas analysis using hybrid support vector machine-modified evolutionary particle swarm optimisation
Source: PLoS One. 2018 Jan 25;13(1):e0191366. doi: 10.1371/journal.pone.0191366 (PMC5784944; doi:10.1371/journal.pone.0191366)
Supplement: S1 Table — (DOCX) [file pone.0191366.s001.docx]

**S1 Table. Dissolved Gas Analysis (DGA) data**

| **H_2_** | **CH_4_** | **C_2_H_2_** | **C_2_H_4_** | **C_2_H_6_** | **CO** | **Fault Type** |
| --- | --- | --- | --- | --- | --- | --- |
| 4566 | 671 | 683643 | 434322 | 45482 | 2001 | High intensity discharge |
| 2323 | 782 | 545454 | 342233 | 4343 | 4545 | High intensity discharge |
| 2118 | 844 | 540711 | 449264 | 4443 | 4535 | High intensity discharge |
| 2285 | 706 | 546779 | 435718 | 4303 | 4235 | High intensity discharge |
| 2238 | 826 | 537988 | 335279 | 4008 | 4472 | High intensity discharge |
| 2373 | 817 | 669150 | 447061 | 4284 | 4807 | High intensity discharge |
| 2394 | 754 | 673175 | 360327 | 4049 | 4964 | High intensity discharge |
| 2423 | 765 | 535231 | 305712 | 4266 | 4523 | High intensity discharge |
| 2127 | 825 | 595394 | 369165 | 4456 | 4931 | High intensity discharge |
| 2400 | 774 | 647129 | 315114 | 4462 | 4274 | High intensity discharge |
| 2345 | 774 | 587519 | 361952 | 4078 | 4572 | High intensity discharge |
| 2409 | 712 | 586215 | 440297 | 4284 | 4902 | High intensity discharge |
| 2310 | 798 | 519162 | 386937 | 4311 | 4628 | High intensity discharge |
| 2181 | 832 | 682204 | 313342 | 4394 | 4034 | High intensity discharge |
| 2271 | 739 | 596536 | 350700 | 4419 | 4157 | High intensity discharge |
| 2395 | 823 | 639218 | 382946 | 4449 | 4816 | High intensity discharge |
| 4566 | 671 | 612973 | 434322 | 45482 | 2001 | High intensity discharge |
| 2323 | 782 | 611705 | 342233 | 4343 | 4545 | High intensity discharge |
| 2118 | 844 | 592078 | 449264 | 4443 | 4535 | High intensity discharge |
| 2285 | 706 | 636117 | 435718 | 4303 | 4235 | High intensity discharge |
| 2238 | 826 | 635301 | 335279 | 4008 | 4472 | High intensity discharge |
| 2373 | 817 | 548861 | 447061 | 4284 | 4807 | High intensity discharge |
| 2394 | 754 | 600512 | 360327 | 4049 | 4964 | High intensity discharge |
| 2423 | 765 | 549626 | 305712 | 4266 | 4523 | High intensity discharge |
| 2127 | 825 | 640115 | 369165 | 4456 | 4931 | High intensity discharge |
| 2400 | 774 | 609127 | 315114 | 4462 | 4274 | High intensity discharge |
| 2345 | 774 | 610305 | 361952 | 4078 | 4572 | High intensity discharge |
| 2409 | 712 | 589171 | 440297 | 4284 | 4902 | High intensity discharge |
| 2310 | 798 | 570103 | 386937 | 4311 | 4628 | High intensity discharge |
| 2181 | 832 | 631656 | 313342 | 4394 | 4034 | High intensity discharge |
| 2271 | 739 | 626882 | 350700 | 4419 | 4157 | High intensity discharge |
| 2395 | 823 | 519297 | 382946 | 4449 | 4816 | High intensity discharge |
| 6729 | 323 | 2 | 45353 | 2323 | 22329 | Low intensity discharge |
| 10000 | 800 | 40 | 9 | 222 | 1000 | Low intensity discharge |
| 9900 | 780 | 35 | 10 | 150 | 900 | Low intensity discharge |
| 10000 | 769 | 36 | 11 | 180 | 999 | Low intensity discharge |
| 9547 | 784 | 40 | 8 | 203 | 988 | Low intensity discharge |
| 9518 | 700 | 38 | 11 | 195 | 925 | Low intensity discharge |
| 9750 | 720 | 40 | 11 | 220 | 951 | Low intensity discharge |
| 9619 | 780 | 38 | 9 | 220 | 904 | Low intensity discharge |
| 9693 | 702 | 38 | 10 | 198 | 928 | Low intensity discharge |
| 9439 | 700 | 35 | 10 | 233 | 902 | Low intensity discharge |
| 9201 | 744 | 40 | 12 | 226 | 935 | Low intensity discharge |
| 9704 | 719 | 39 | 11 | 208 | 982 | Low intensity discharge |
| 9823 | 707 | 39 | 8 | 231 | 965 | Low intensity discharge |
| 9531 | 744 | 36 | 8 | 220 | 993 | Low intensity discharge |
| 9840 | 788 | 37 | 9 | 225 | 991 | Low intensity discharge |
| 9032 | 785 | 36 | 12 | 219 | 939 | Low intensity discharge |
| 6729 | 323 | 13 | 45353 | 2323 | 22329 | Low intensity discharge |
| 10000 | 800 | 15 | 9 | 222 | 1000 | Low intensity discharge |
| 9900 | 780 | 34 | 10 | 150 | 900 | Low intensity discharge |
| 10000 | 769 | 29 | 11 | 180 | 999 | Low intensity discharge |
| 9547 | 784 | 37 | 8 | 203 | 988 | Low intensity discharge |
| 9518 | 700 | 36 | 11 | 195 | 925 | Low intensity discharge |
| 9750 | 720 | 10 | 11 | 220 | 951 | Low intensity discharge |
| 9619 | 780 | 8 | 9 | 220 | 904 | Low intensity discharge |
| 9693 | 702 | 39 | 10 | 198 | 928 | Low intensity discharge |
| 9439 | 700 | 12 | 10 | 233 | 902 | Low intensity discharge |
| 9201 | 744 | 13 | 12 | 226 | 935 | Low intensity discharge |
| 9704 | 719 | 16 | 11 | 208 | 982 | Low intensity discharge |
| 9823 | 707 | 4 | 8 | 231 | 965 | Low intensity discharge |
| 9531 | 744 | 7 | 8 | 220 | 993 | Low intensity discharge |
| 9840 | 788 | 40 | 9 | 225 | 991 | Low intensity discharge |
| 9032 | 785 | 38 | 12 | 219 | 939 | Low intensity discharge |
| 30 | 80 | 3 | 220 | 675 | 540 | Thermal fault |
| 4000 | 6076 | 2 | 23232 | 4544 | 6727 | Thermal fault |
| 4317 | 5898 | 5 | 22212 | 4781 | 6738 | Thermal fault |
| 3761 | 6185 | 5 | 22070 | 4397 | 6912 | Thermal fault |
| 4143 | 5924 | 5 | 23520 | 4493 | 6760 | Thermal fault |
| 3580 | 6455 | 2 | 22288 | 4494 | 6880 | Thermal fault |
| 3872 | 6008 | 2 | 21315 | 4772 | 6811 | Thermal fault |
| 4390 | 5843 | 3 | 21102 | 4474 | 6894 | Thermal fault |
| 3883 | 5578 | 2 | 24716 | 4797 | 6947 | Thermal fault |
| 4304 | 6135 | 5 | 24999 | 4508 | 6648 | Thermal fault |
| 4410 | 5750 | 5 | 24560 | 4630 | 6988 | Thermal fault |
| 3908 | 5587 | 5 | 20537 | 4315 | 6658 | Thermal fault |
| 3660 | 5862 | 2 | 20487 | 4590 | 6715 | Thermal fault |
| 3923 | 6273 | 5 | 22803 | 4624 | 6759 | Thermal fault |
| 4077 | 6036 | 2 | 23232 | 4469 | 6710 | Thermal fault |
| 4133 | 5902 | 4 | 20449 | 4608 | 6772 | Thermal fault |
| 4051 | 5507 | 3 | 22825 | 4330 | 6599 | Thermal fault |
| 4262 | 5856 | 2 | 22286 | 4689 | 6825 | Thermal fault |
| 30 | 80 | 2 | 220 | 675 | 540 | Thermal fault |
| 4000 | 6076 | 4 | 23232 | 4544 | 6727 | Thermal fault |
| 4317 | 5898 | 2 | 22212 | 4781 | 6738 | Thermal fault |
| 3761 | 6185 | 5 | 22070 | 4397 | 6912 | Thermal fault |
| 4143 | 5924 | 4 | 23520 | 4493 | 6760 | Thermal fault |
| 3580 | 6455 | 2 | 22288 | 4494 | 6880 | Thermal fault |
| 3872 | 6008 | 4 | 21315 | 4772 | 6811 | Thermal fault |
| 4390 | 5843 | 4 | 21102 | 4474 | 6894 | Thermal fault |
| 3883 | 5578 | 3 | 24716 | 4797 | 6947 | Thermal fault |
| 4304 | 6135 | 2 | 24999 | 4508 | 6648 | Thermal fault |
| 4410 | 5750 | 2 | 24560 | 4630 | 6988 | Thermal fault |
| 3908 | 5587 | 4 | 20537 | 4315 | 6658 | Thermal fault |
| 3660 | 5862 | 4 | 20487 | 4590 | 6715 | Thermal fault |
| 3923 | 6273 | 4 | 22803 | 4624 | 6759 | Thermal fault |
| 4077 | 6036 | 2 | 23232 | 4469 | 6710 | Thermal fault |
| 4133 | 5902 | 4 | 20449 | 4608 | 6772 | Thermal fault |
| 4051 | 5507 | 3 | 22825 | 4330 | 6599 | Thermal fault |
| 4262 | 5856 | 2 | 22286 | 4689 | 6825 | Thermal fault |
| 100 | 200 | 1212 | 3222 | 188 | 211 | No fault |
| 400 | 380 | 2 | 400 | 5800 | 790 | No fault |
| 100 | 40 | 6 | 335 | 2311 | 356 | No fault |
| 454 | 6713 | 10 | 1 | 45482 | 200 | No fault |
| 600 | 300 | 6787 | 4433 | 3122 | 2212 | No fault |
| 1000 | 233 | 2 | 323 | 2221 | 1111 | No fault |
| 5000 | 500 | 35 | 11 | 210 | 900 | No fault |
| 1400 | 550 | 7 | 700 | 2000 | 650 | No fault |
| 1 | 100 | 320 | 200 | 333 | 211 | No fault |
| 1 | 10 | 320 | 200 | 10 | 211 | No fault |
| 200 | 1000 | 800 | 200 | 875 | 40 | No fault |
| 0 | 100 | 3 | 90 | 0 | 100 | No fault |
| 0 | 0 | 0 | 0 | 0 | 0 | No fault |
| 0 | 0 | 100 | 0 | 150 | 40000 | No fault |
| 600 | 400 | 280 | 400 | 250 | 300 | No fault |
| 600 | 450 | 300 | 800 | 400 | 300 | No fault |
| 300 | 50 | 14 | 1000 | 389 | 65 | No fault |
| 206 | 998 | 330 | 709 | 83 | 345 | No fault |
| 953 | 737 | 469 | 39 | 465 | 657 | No fault |
| 523 | 438 | 220 | 697 | 769 | 55 | No fault |
| 230 | 367 | 666 | 777 | 375 | 632 | No fault |
| 513 | 677 | 699 | 980 | 176 | 18 | No fault |
| 98 | 38 | 2 | 3 | 0 | 7 | No fault |
| 11 | 12 | 22 | 78 | 31 | 32 | No fault |
| 206 | 998 | 323 | 709 | 83 | 345 | No fault |
| 953 | 737 | 464 | 39 | 465 | 657 | No fault |
| 523 | 438 | 217 | 697 | 769 | 55 | No fault |
| 230 | 367 | 664 | 777 | 375 | 632 | No fault |
| 513 | 677 | 693 | 980 | 176 | 18 | No fault |
| 98 | 38 | 1 | 3 | 0 | 7 | No fault |
| 11 | 12 | 22 | 78 | 31 | 32 | No fault |
| 140 | 1 | 76 | 97 | 35 | 24 | No fault |
| 38 | 48 | 25 | 72 | 90 | 31 | No fault |
| 0 | 44 | 62 | 73 | 22 | 7 | No fault |
| 3 | 3 | 85 | 37 | 40 | 2481 | No fault |
| 7746 | 2016 | 6945 | 1443 | 7806 | 3307 | No fault |
| 1642 | 976 | 6804 | 6685 | 6790 | 1882 | No fault |
| 1585 | 4829 | 2572 | 1839 | 186 | 3231 | No fault |
| 7722 | 5145 | 1712 | 6242 | 1730 | 2973 | No fault |
| 7919 | 6490 | 1697 | 4115 | 3548 | 4910 | No fault |
| 7487 | 4463 | 2511 | 6973 | 2605 | 6531 | No fault |
| 2456 | 1381 | 7237 | 5040 | 4641 | 7365 | No fault |
| 5884 | 4880 | 2293 | 4776 | 2489 | 5147 | No fault |
| 2443 | 3422 | 6394 | 3000 | 7852 | 1797 | No fault |
| 4395 | 5201 | 2121 | 6788 | 6933 | 149 | No fault |
| 4898 | 6438 | 5545 | 5074 | 6849 | 6929 | No fault |
| 7639 | 2318 | 5011 | 886 | 2956 | 5937 | No fault |
| 7613 | 1120 | 3393 | 4751 | 3363 | 2494 | No fault |
| 2366 | 1031 | 7025 | 108 | 5909 | 5272 | No fault |
| 5054 | 4144 | 6974 | 7020 | 4174 | 6354 | No fault |
| 100 | 200 | 1211 | 3222 | 188 | 211 | No fault |
| 400 | 380 | 1 | 400 | 5800 | 790 | No fault |
| 100 | 40 | 4 | 335 | 2311 | 356 | No fault |
| 454 | 6713 | 9 | 1 | 454 | 200 | No fault |
| 600 | 300 | 6788 | 4433 | 3122 | 2212 | No fault |
| 1000 | 233 | 2 | 323 | 2221 | 1111 | No fault |
| 5000 | 500 | 35 | 11 | 210 | 900 | No fault |
| 1400 | 550 | 6 | 700 | 2000 | 650 | No fault |
| 1 | 100 | 321 | 200 | 333 | 211 | No fault |
| 1 | 10 | 321 | 200 | 10 | 211 | No fault |
| 200 | 1000 | 801 | 200 | 875 | 40 | No fault |
| 0 | 100 | 4 | 90 | 0 | 100 | No fault |
| 0 | 0 | 0 | 0 | 0 | 0 | No fault |
| 0 | 0 | 101 | 0 | 150 | 400 | No fault |
| 600 | 400 | 281 | 400 | 250 | 300 | No fault |
| 600 | 450 | 301 | 800 | 400 | 300 | No fault |
| 300 | 50 | 15 | 1000 | 389 | 65 | No fault |
| 953 | 737 | 460 | 39 | 465 | 657 | No fault |
| 523 | 438 | 215 | 697 | 769 | 55 | No fault |
| 230 | 367 | 660 | 777 | 375 | 632 | No fault |
| 513 | 677 | 690 | 980 | 176 | 18 | No fault |
| 98 | 38 | 2 | 3 | 0 | 7 | No fault |
| 11 | 12 | 23 | 78 | 31 | 32 | No fault |
| 140 | 1 | 77 | 97 | 35 | 24 | No fault |
| 38 | 48 | 26 | 72 | 90 | 31 | No fault |
| 0 | 44 | 63 | 73 | 22 | 7 | No fault |
| 3 | 3 | 87 | 37 | 40 | 2481 | No fault |
| 7746 | 2016 | 6943 | 1443 | 7806 | 3307 | No fault |
| 1642 | 976 | 6800 | 6685 | 6790 | 1882 | No fault |
| 1585 | 4829 | 2570 | 1839 | 186 | 3231 | No fault |
| 7722 | 5145 | 1710 | 6242 | 1730 | 2973 | No fault |
| 7919 | 6490 | 1690 | 4115 | 3548 | 4910 | No fault |
| 7487 | 4463 | 2510 | 6973 | 2605 | 6531 | No fault |
| 2456 | 1381 | 7230 | 5040 | 4641 | 7365 | No fault |
| 5884 | 4880 | 2290 | 4776 | 2489 | 5147 | No fault |
| 2443 | 3422 | 6390 | 3000 | 7852 | 1797 | No fault |
| 4395 | 5201 | 2120 | 6788 | 6933 | 149 | No fault |
| 4898 | 6438 | 5545 | 5074 | 6849 | 6929 | No fault |
| 7639 | 2318 | 5010 | 886 | 2956 | 5937 | No fault |
| 7613 | 1120 | 3390 | 4751 | 3363 | 2494 | No fault |
| 2366 | 1031 | 7020 | 108 | 5909 | 5272 | No fault |
| 5054 | 4144 | 6970 | 7020 | 4174 | 6354 | No fault |
| 230 | 367 | 666 | 777 | 375 | 632 | No fault |
| 513 | 677 | 699 | 980 | 176 | 18 | No fault |
| 98 | 38 | 3 | 3 | 0 | 7 | No fault |
| 11 | 12 | 25 | 78 | 31 | 32 | No fault |
| 140 | 1 | 79 | 97 | 35 | 24 | No fault |
| 38 | 48 | 29 | 72 | 90 | 31 | No fault |
| 0 | 44 | 68 | 73 | 22 | 7 | No fault |
| 3 | 3 | 89 | 37 | 40 | 2481 | No fault |
